# Development of a bioavailability‐based risk assessment framework for nickel in Southeast Asia and Melanesia

**DOI:** 10.1002/ieam.4384

**Published:** 2021-02-08

**Authors:** Emily R Garman, Christian E Schlekat, Ellie Middleton, Graham Merrington, Adam Peters, Ross Smith, Jenny L Stauber, Kenneth MY Leung, Francesca Gissi, Monique T Binet, Merrin S Adams, Megan L Gillmore, Lisa A Golding, Dianne Jolley, Zhen Wang, Amanda Reichelt‐Brushett

**Affiliations:** ^1^ NiPERA Inc. Durham North Carolina USA; ^2^ wca, Faringdon, Oxon United Kingdom; ^3^ Hydrobiology Brisbane Australia; ^4^ CSIRO Land and Water Lucas Heights New South Wales Australia; ^5^ State Key Laboratory of Marine Pollution and Department of Chemistry, City University of Hong Kong Kowloon Hong Kong China; ^6^ CSIRO, Oceans and Atmosphere Lucas Heights New South Wales Australia; ^7^ School of Earth, Atmosphere and Life Sciences, University of Wollongong New South Wales Australia; ^8^ Institute of Marine Sciences and Guangdong Provincial Key Laboratory of Marine Biotechnology, Shantou University Shantou China; ^9^ Marine Ecology Research Centre, School of Environment, Science and Engineering, Southern Cross University Lismore New South Wales Australia

**Keywords:** Nickel, Bioavailability‐based approaches, Metals risk assessment, Tropical risk assessment

## Abstract

Nickel laterite ore deposits are becoming increasingly important sources of Ni for the global marketplace and are found mainly in tropical and subtropical regions, including Indonesia, the Philippines, Papua New Guinea, Cuba, and New Caledonia. There are few legislatively derived standards or guidelines for the protection of aquatic life for Ni in many of these tropical regions, and bioavailability‐based environmental risk assessment (ERA) approaches for metals have mainly been developed and tested in temperate regions, such as the United States and Europe. This paper reports on a multi‐institutional, 5‐y testing program to evaluate Ni exposure, effects, and risk characterization in the Southeast Asia and Melanesia (SEAM) region, which includes New Caledonia, Papua New Guinea, the Philippines, and Indonesia. Further, we have developed an approach to determine if the individual components of classical ERA, including effects assessments, exposure assessments, and risk characterization methodologies (which include bioavailability normalization), are applicable in this region. A main conclusion of this research program is that although ecosystems and exposures may be different in tropical systems, ERA paradigms are constant. A large chronic ecotoxicity data set for Ni is now available for tropical species, and the data developed suggest that tropical ecosystems are not uniquely sensitive to Ni exposure; hence, scientific support exists for combining tropical and temperate data sets to develop tropical environmental quality standards (EQSs). The generic tropical database and tropical exposure scenarios generated can be used as a starting point to examine the unique biotic and abiotic characteristics of specific tropical ecosystems in the SEAM region. *Integr Environ Assess Manag* 2021;17:802–813. © 2021 The Authors. *Integrated Environmental Assessment and Management* published by Wiley Periodicals LLC on behalf of Society of Environmental Toxicology & Chemistry (SETAC)

## KEY POINTS

- An approach to determine if the individual components of classical environmental risk assessment (ERA) methodologies are applicable in the Southeast Asia and Melanesia (SEAM) region is presented.

- A main conclusion is that, although ecosystems and exposures may be different in tropical systems, ERA paradigms are constant.

- A large chronic ecotoxicity data set for Ni is now available for tropical species, and the data developed suggest that tropical ecosystems are not uniquely sensitive to Ni exposure.

- The generic tropical database and tropical exposure scenarios generated can be used as a starting point to examine the unique biotic and abiotic characteristics of specific tropical ecosystems in the SEAM region.

## INTRODUCTION

Bioavailability‐based risk assessment approaches for metals are globally recognized as the state‐of‐the‐science for conducting environmental risk assessments (ERAs) and developing environmental quality standards (EQSs) (EU 2013; ANZG 2018; Schlekat et al. 2020). These frameworks rely on bioavailability models, such as the Biotic Ligand Model (BLM) (Adams et al. 2020), many of which have been developed and extensively tested in temperate regions and are used within existing legislations such as European Union's (EU's) Registration, Evaluation, Authorisation and Restriction of Chemicals (REACH) (EC 2007) and Water Framework Directive (EU 2013). However, direct application of these models and ERA approaches to metal‐producing regions outside of these areas (i.e., tropical and subtropical regions) can be uncertain without first evaluating the impact that the differences in geochemistry, animal physiology and life history (Peters et al. 2018), and the taxonomic composition of endemic ecosystems may have on the validity of the ERA frameworks and models. In Ni‐producing regions such as Southeast Asia and Melanesia (SEAM), naturally enriched geology, mining, smelting, and refining can lead to high Ni exposures, and putting these values into an environmentally relevant context is difficult without region‐specific risk assessment tools. There are few legislatively derived standards or guidelines for the protection of aquatic life in the SEAM region for Ni, unlike some areas in the world, for example, Australia, North America, and Europe (USEPA 1985; EU 2013; ANZG 2018).

Nickel laterite ore deposits are becoming increasingly important sources of Ni for the global marketplace and now account for 60% of the world's Ni supply (Mudd 2010). These ore bodies are found mainly in tropical and subtropical regions, including Indonesia, the Philippines, Cuba, Papua New Guinea (PNG), and New Caledonia (Dalvi et al. 2004; Mudd 2010; Mudd and Jowitt 2014). The mining, refining, and smelting of Ni laterites can lead to solubilization of not only Ni but also co‐occurring elements such as Co, Cr, Mn, sulfate, and chloride and their subsequent release into waters, sediments, and soils (Fernandez et al. 2006). Although Ni is just 1 environmental stressor associated with Ni operations in SEAM, it is the primary focus in this ERA framework.

Tropical ecosystems differ from temperate ecoregions in that they may have different climate and geochemistry, unique ecosystem composition, and generally greater biodiversity. Remote locations and extreme weather conditions such as episodic events of high rainfall during summer monsoon or typhoon can make these areas difficult operational environments. Consequently, there is a paucity of data on exposure and ecotoxicological effects of Ni in tropical marine and fresh waters and sediments (Gissi et al. 2016; Binet et al. 2018), very few data exist on the influence of water quality on Ni toxicity in tropical regions (Gissi et al. 2015), and there are no data on mechanisms of Ni toxicity in tropical regions. The combination of increased Ni production, potentially sensitive tropical ecosystems, and uncertainties about the protectiveness of current guidelines primarily derived from temperate species for safeguarding these tropical ecosystems creates an urgent need to develop appropriate ERA approaches for these environments.

Traditional ERA approaches give a clear framework for the collection, organization, evaluation, assessment, and characterization of data to deliver information on the form and magnitude of potential chemical risks. Using such a framework (e.g., USEPA 1992) and focusing upon each element of the ERA paradigm provides an opportunity for targeted refinement and greater ecological realism in the assessment (e.g., Omenn 1995). The use of this framework provides a common thread through the challenges that are typical to many ERAs, and also identifies logical stopping points in the process where progress can be assessed. In order to use these approaches in regions where they have not been developed or extensively tested, it is critical to validate the bioavailability‐based risk assessment approaches to ensure that their application remains appropriate in these environments (Garman et al. 2020). The validation process looks different for each region and is predicated on the examination of the unique aspects, particularly the local geochemistry, flora, and fauna, of each region, that could impact the assessment and tools. The aim of the present research was to develop a bioavailability‐based ERA approach and assess the validity of its use in a region outside of which the tools and approaches have been developed, using an extensive research program on Ni risk assessment in the tropical SEAM region as an example.

## METHODOLOGY

**Figure 1:**
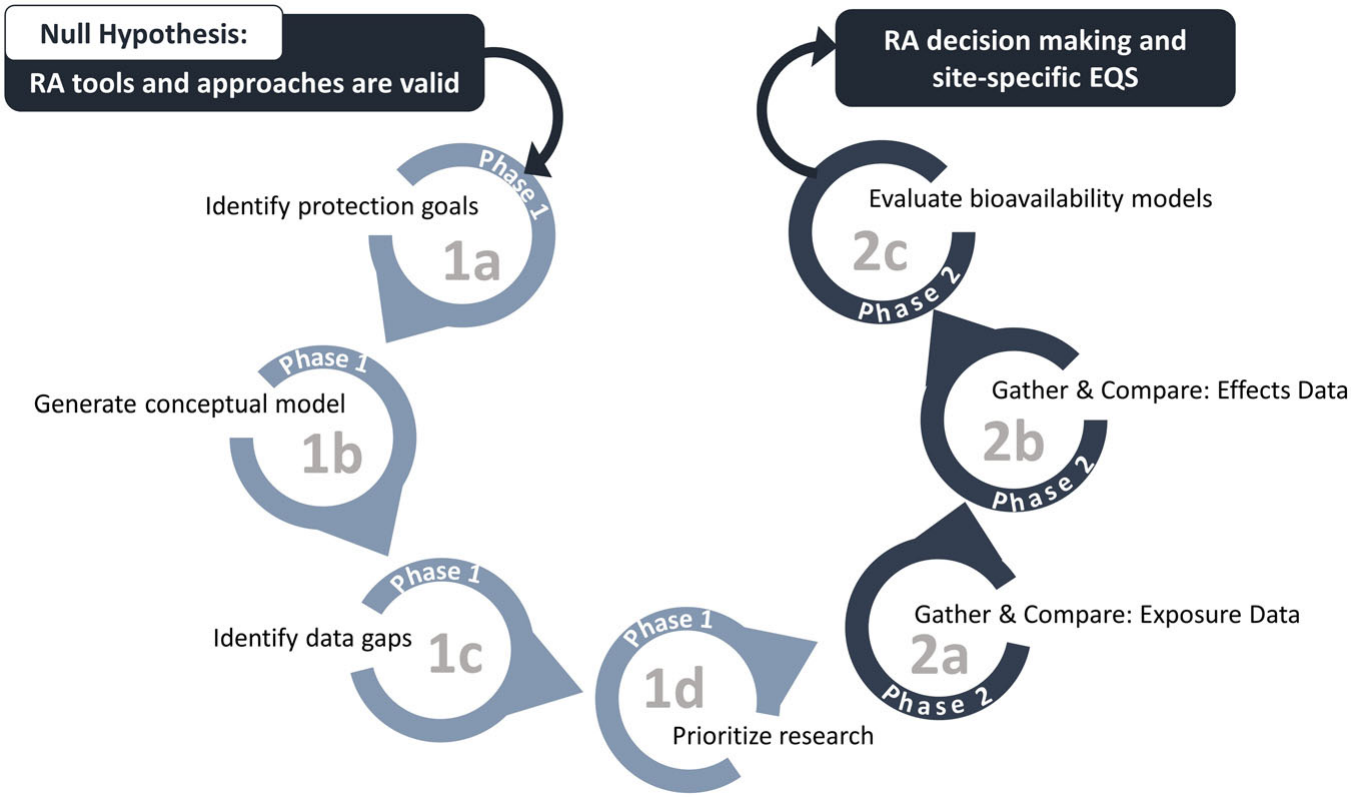
Phased approach for building blocks of environmental risk assessment (ERA) approach. EQS = environmental quality standard; ERA = environmental risk assessment; RA =  risk assessment.

We developed an approach to determine the applicability of individual components of classical ERA, including effects assessments, exposure assessments, and risk characterization methodologies (which includes bioavailability normalization) in the tropical region. We attempted to examine if risk assessment approaches can be directly applied in the region of interest or if there are no conceptual differences that would render these approaches invalid. We then set out to disprove the null hypothesis. The first step was to identify the unique characteristics that could impact the risk assessment approaches and investigate their influence. We used several approaches to identify the most important “differences,” such as the identification of protection goals, development of a conceptual model, data gap analysis, and research prioritization (Figure 1). Based on the outcome of these prioritization steps, we then conducted research on the fate and transport of metals in tropical environments and ecotoxicity data in tropical water and sediment ecosystems to facilitate the translation of refined ERA approaches used globally to the SEAM region. By demonstrating that the constructs of traditional ERA can be used in tropical ecosystems, we have developed the building blocks of a tiered approach that can be used for performing ERA in the SEAM region (Figure 1).

### Phase 1: Research program development and prioritization

#### Problem formulation

Currently, there are few regulatory requirements related to the protection of aquatic life against Ni in the SEAM region. Indonesia, Malaysia, the Philippines, and Thailand have regulations based on classes of water and their uses (Supporting Information SI1), whereas PNG has generic standards and a code of practice for the mining industry for receiving waters and there is a multinational standard adopted by all countries in the Association of Southeast Asian Nations (ASEAN) for marine ecosystems. However, these do not explicitly account for bioavailability, and locally relevant protection limit values of Ni for use in ERA are not readily available. Most industrial facilities in the region aim to comply with limits that have been derived based on human health protection goals, for example, drinking water standards. Standards for the protection of fish, which may be aimed at the protection of important food sources, are also applied in some regions (e.g., Japan [MOE 2018]). These standards are likely to be of low relevance to the environment, especially for Ni, because the most sensitive receptors for Ni have been shown to be aquatic invertebrates (Schlekat et al. 2010; Gissi et al. 2018; Peters et al. 2018).

One of the main difficulties in determining the appropriateness of regulatory approaches was the absence of reliable ecotoxicological data for tropical species with which we could confidently assess risk. In 2014, chronic ecotoxicity data for Ni were available for 17 marine species, but only one of these species inhabited tropical waters (DeForest and Schlekat 2013). Similarly, the freshwater ecotoxicity database for Ni that had been accepted for regulatory purposes in Europe was the most comprehensive and ecologically diverse; however, the species comprising this database were principally from temperate regions (EC 2008). The clear conclusion during the problem formulation phase was that additional ecotoxicological data were required to confidently identify the appropriate protection goals, unique habitats, and important ecosystems for consideration in the SEAM region.

It was also necessary to identify appropriate ecosystem protection goals to apply within the ERA framework that provided sufficient flexibility to be appropriate to a variety of different land uses. For the present work we considered the broad “levels of protection” detailed in the recently developed online Australian and New Zealand Guidelines (ANZG) for Fresh and Marine Water Quality (ANZG 2018). An attribute of these guidelines is the matching of the protection level to a water body based on its ecosystem condition, that is, its current or desired ecosystem condition relative to the degree of human disturbance. There are 3 categories of condition with different levels of protection: high conservation or ecological value systems requiring protection of 99% of species from toxicants, slightly to moderately disturbed systems requiring 95% species protection, and highly disturbed systems requiring at least 80% to 90% species protection from toxicants. In the present assessment, the relevant protection level is determined by the exposure scenario considered.

#### Development of conceptual model

Building a conceptual model of contaminant sources and exposure pathways is generally accepted as a routine starting point for any ERA (e.g., USEPA 1992) and was also a critical step for the present research initiative. The conceptual model begins a cascade of steps in the research process: identification of where exposures are likely to occur and the exposed ecosystems, prioritization of ecosystems, determination of availability of effects data for exposed ecosystems, et cetera.

Nickel exposures in the SEAM region result from Ni mining, smelting, and refining, and often occur adjacent to the coast because the vast majority of Ni ores, concentrates, and cathodes are transported by ship to overseas markets. From these operations, dissolved and particulate Ni can be released via direct tailings discharge, site runoff, seepage from waste storage facilities, runoff and seepage from the disposal or storage sites for waste materials, via solids or slurries from the emission abatement systems, or as dust from stack and fugitive emissions (Mudd 2010; Reichelt‐Brushett 2012).

**Figure 2:**
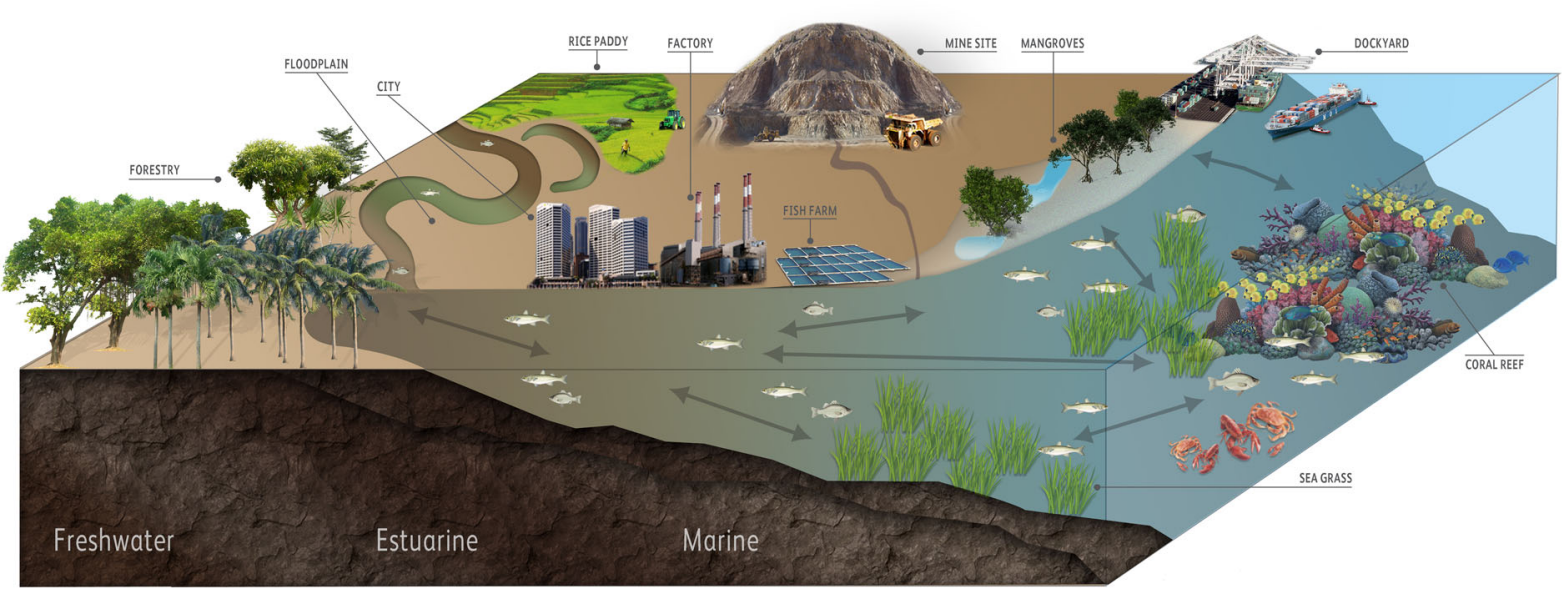
Generic conceptual model of Southeast Asia and Melanesia region. TERAP = Tropical Environmental Risk Assessment of Nickel.

From the generic conceptual model (Figure 2), it can be seen that the potential receiving environments for Ni are likely to be fresh, estuarine, marine, and coastal waters, including potentially sensitive, critical, and biodiverse assemblages such as mangrove, seagrass, and coral ecosystems. Exposures to benthic communities are likely to occur due to runoff of particulate matter that result from intense precipitation events. Our understanding of these environments and the ecological assemblages present therein are less well developed than the temperate systems where ERA understanding has been developed (e.g., ECHA 2016). Our focus was initially on aquatic and benthic habitats because they are where the Ni exposures are most likely to occur.

Among the most important considerations when assessing impacts of laterite mining is the potential sensitivity of the receiving habitats. Many ecosystems of the SEAM region are unique in their key structural components, the patterns of interconnectedness of these habitats, and the high levels of biodiversity that they support (Bouchet et al. 2002; Roberts et al. 2002) (Figure 2). Maintaining connectivity of tropical freshwater, estuarine, and marine systems is particularly important for many migratory species (Nemeth 2009), which are key components of ecosystems in this region (Keith et al. 2008). These species include endemic amphidromous fishes and shrimps, as well as catadromous fishes and eels, which move between freshwater and marine systems at various stages of their life cycle.

#### Data gap analysis

From previous experiences of regional (e.g., Van Sprang et al. 2009) and global ERAs of metals (e.g., Marks et al. 2017), a commonly encountered challenge is the paucity of relevant, high‐quality exposure data. The use of scenario‐based approaches, that is, focusing upon ecosystems for which data exist, represents an appropriate starting position and fits with the premise of the ERA paradigm and specifically focuses efforts of refinement (e.g., Daam and Van den Brink 2010). Current scientific understanding of the behavior and fate of Ni in aquatic ecosystems shows the need to account for bioavailability in order to deliver a robust ERA (Merrington et al. 2016; Buxton et al. 2019). Therefore, to incorporate bioavailability in a scenario‐based approach, data on the water chemistry parameters that affect Ni bioavailability are also required.

Several national regulatory jurisdictions have specific geographical requirements for the use of specific data, based on the view that the sensitivity of organisms to chemical exposures may be linked to temperature and/or local species specificity (USEPA 1985; CCME 2007). Robust evidence to support this view is very limited. Wang et al. (2014) found only small differences in the acute toxicity of chemicals between tropical and temperate marine biota, and these differences may be confounded by the different temperatures and salinities of the tests. In another study reviewing evidence of the differences between temperate and tropical freshwater ecosystems for the ERA of pesticides, Daam and Van den Brink (2010) identified data that show both less and more sensitivity of effects in tropical exposure scenarios compared to temperate equivalents. The authors considered the testing of large numbers of tropical species from a large number of ecological communities to be practically and financially unfeasible. The question in the early stages of this current assessment was therefore focused on the scientific validity of applying the very extensive chronic ecotoxicity database for Ni (EC 2008) containing species from tropical and temperate ecosystems to the SEAM region (See section *Phase 2: Research and data collection: Effects assessment*).

Data gaps were identified based on the existing spread of available data across taxa, with particular focus on ensuring that there were sufficient high‐quality chronic data, their ecological relevance to SEAM, and the sensitivity of particular taxa based on other Ni studies from temperate or tropical marine or freshwater studies. A further literature search was undertaken to identify the ecological importance of key taxa to the SEAM region, based on endemism and biodiversity (Gissi et al. 2016; Binet et al. 2018). The literature search was used to prioritize future testing to obtain Ni toxicity data for tropical species from this region.

#### Research prioritization

The research prioritization step is important in emphasizing research that is both impactful and achievable, and several methods are available to do this. Throughout the Nickel Tropical Research Program, research prioritization was evaluated and refined to ensure that the appropriate questions were addressed. Two potential methods that can be used for this purpose are a modified Leopold matrix and a sensitivity analysis approach. Both can be utilized to quantitatively evaluate the impact of additional data points on statistically derived toxicity estimates. Although these steps were not initially used in the research prioritization phase of the present program, they were utilized at later stages to ensure the prioritization of future research. In hindsight, these approaches could have been applied in phase 1 of the SEAM program and will be used in continued research efforts in these areas.

The Leopold matrix is a quantitative environmental impact assessment method pioneered in 1971 (Leopold et al. 1971). In the context of ERA, the Leopold matrix can be employed to evaluate and prioritize research needs in a specific region and identify gaps in knowledge for effects assessment of important habitat types and biogeochemical processes that control Ni bioavailability. A modified Leopold matrix based on uncertainty, importance, and practicality can be used to prioritize research that is both impactful and achievable.

The Leopold matrix approach relies on expert opinion to identify environmental compartments with gaps and to rank them accordingly on a scale of 1 to 5 for the following:

- amount of certainty and data (focus on ecotoxicology) availability (1 = lowest and 5 = highest),
- relative importance and impact (1 = lowest, 5 = highest), and
- practicality (1 = hardest and 5 = easiest).

The sum of the 3 assessment scores can be used to rank priorities for research needs. This approach can be used at the beginning of the research phase to prioritize data collection in relation to the gaps identified in the prior step and could be repeated at the end of the research program to develop additional research needs or identify future research directions. An example of a Leopold matrix for future tropical risk assessment research is presented in the Supporting Information (SI2).

Additionally, a sensitivity analysis approach can be utilized to quantitatively evaluate the impact of additional data points on statistically derived toxicity estimates (e.g., hazardous concentration [HC] that adversely affects 5% or 50% of species, i.e., HC5 or HC50 values). The sensitivity of an existing species sensitivity distribution (SSD), and particularly thresholds derived from it such as the HC5 value, to additional ecotoxicity data could be assessed by the addition of hypothetical data to the data set and replotting the SSD (e.g., Wheeler et al. 2002; Wang et al. 2008). The hypothetical data used could be generated randomly, guided by the distribution of values already available, or could be selected arbitrarily. By replotting the updated SSD with a variety of additional data included, it would be possible to assess the potential impact of performing additional testing on an existing SSD. This approach would provide an indication of how many additional tests would be required, and the range of sensitivities that they covered, in order to affect the resulting HC5 value from the SSD by a predetermined amount. Taken together, the conceptual model, data gap analysis, and research prioritization provide a roadmap for *Phase 2: Research and data collection*.

### Phase 2: Research and data collection

#### Exposure assessment

Generic emission and exposure scenarios can be developed for typical mining scenarios, which cover the most likely routes by which metals may be released into the local environment. Generic emission scenarios are likely to utilize worst‐case estimates of emission factors and consequently involve very large uncertainties. Although many operations will show differences, the use of worst‐case values should ensure that any uncertainty causes the resulting exposure estimates to be higher than they occur in reality, so will tend to overestimate any potential environmental risks and result in a very precautionary assessment (ECHA 2016). When risk is shown in such precautionary assessments, this should trigger the collection of additional site‐specific information that can be used to refine the assessment and develop a more relevant determination of risk.

The general goal for improving site‐specific exposure assessments is to improve accuracy in calculating the emissions from the site and understand how they are released into the environment. It is important to include all relevant sources of emissions, bearing in mind that some of the sources may be diffuse, such as leaching from stockpiled raw materials or stored waste materials such as tailings. Improving the accuracy of predicted releases into the environment usually takes account of the relative flow rates of point sources and the receiving waters in order to calculate the level of dilution available. Likewise, it may be necessary to make similar estimates of the potential for dilution of emissions from more diffuse sources, which could reach surface waters via leaching from soils or by direct deposition of airborne particulate materials.

The importance of water chemistry constituents, namely pH, hardness, and dissolved organic carbon (DOC), on Ni toxicity in freshwater organisms is well established (e.g., Nys et al. 2016; Buxton et al. 2019) and has been demonstrated for tropical as well as temperate species (Peters et al. 2018). Implementing bioavailability normalization in ERA dictates that relevant key water chemistry data are available. To collect and collate monitoring data for fresh and marine waters within the SEAM region, local regulators, industry groups, and consultants were contacted. The amount and spatial coverage of useable, verifiable monitoring data of key water chemistry determinants (e.g., dissolved Ni concentrations, DOC, water hardness, pH) were limited (see Supporting Information SI3), primarily due to the general lack of broad‐scale government monitoring programs in the region. Based on the geographically limited data set that we compiled, we operationally defined specific bioavailability scenarios that could be used as starting points for site operations that were located in the same geographic region. This does not mean that there are no data, but that these data, although representing a number of extensive local monitoring site networks, several of which had extensive time series, represent patchy coverage of specific ecosystems, site operations, and locations within the region.

Exposure scenarios were not considered for the marine data set, given that there are only small variations in water chemistry parameters (i.e., pH, salinity) in tropical marine ecosystems. It is possible that DOC may influence Ni speciation and bioavailability in marine waters (Blewett et al. 2018). However, very few data were available in the data set we compiled for the region. Therefore, exposure scenario assessments were focused on freshwater systems in the present study.

#### Freshwater exposure scenarios

In order to drive the exposure assessment forward, we have taken these spatially isolated data sets to represent local scenarios within the region. Such an approach is often used in regulatory ERAs (e.g., EMEA 2006) where there are few data, often within a tiered approach that begins with precautionary generic assumptions in a screening‐level assessment, and that triggers data‐gathering efforts when necessary. The scenarios are linked to a number of geological conditions that are relevant to the region and cover volcanic, limestone, and lateritic geology. Volcanic scenarios were further split into circumneutral and high‐pH water types. Multiple scenarios are used for most of these sets of geological conditions, depending upon their relative importance and the observed variation in water chemistries within the available data set.

A selection of water chemistries, which encompassed the range of pH and water hardness characteristics of the data set, have been chosen (Table 1). The scenarios cover a pH range between 7.2 and 8.2, DOC concentrations between 0.5 and 6.6 mg/L, Ca concentrations between 15 and 63 mg/L, and Mg concentrations between 2.7 and 6.7 mg/L. The selection of appropriate water chemistries for the scenario development has tended toward waters with lower concentrations of DOC than may be typical, meaning that the assessments performed will tend to be slightly conservative. Some waters appear to exhibit higher DOC concentrations, so they may be less sensitive than these examples.

**Table 1:** Water chemistries and Ni sensitivities of “typical” Southeast Asia and Melanesia (SEAM) fresh waters (full set of values provided in Supporting Information SI3)

Water	pH	DOC	Ca	Mg	HC5	HC10	HC25
		mg/L	mg/L	mg/L	µg Ni/L	µg Ni/L	µg Ni/L
Volcanic neutral pH #1	7.2	3.9	28.7	4.1	9.4	14.4	29.3
Volcanic neutral pH #2	7.3	6.6	23.5	3.7	11.7	17.5	34.0
Limestone	7.4	2.3	62.8	6.7	8.8	14.1	30.8
Volcanic high pH	7.8	1.5	17.0	3.0	3.3	5.2	11.4
Laterite med DOC, high pH	8.2	1.5	20.9	3.4	1.6	2.8	6.8
Laterite low DOC, high pH	8.2	0.5	25.4	3.8	2.4	4.0	9.0
Laterite high DOC, high pH	8.2	2.4	27.0	4.0	3.4	5.5	12.1

HC = hazardous concentration.

Higher pH waters are likely to be sensitive water types for Ni, due to the generally low water hardness and low DOC concentrations (Nys et al. 2016). The waters in Table 1 can be broadly split between those with a pH below 7.5, which tend to be less sensitive, and those with a pH above 7.5, which tend to be more sensitive. In many surface‐water systems, covariation is observed between pH and hardness, with higher pH waters tending to have higher hardness (Zielhuis and Haring 1981), although this does not particularly appear to be the case in the SEAM waters.

A range of different water chemistry scenarios is required in order for the differences in Ni sensitivity that occur in different surface waters to be represented. An approach that is based on only the most sensitive water chemistries would provide a conservative assessment but would considerably overestimate the potential risks due to Ni at large numbers of sites. A number of different threshold values have been calculated for each of the individual water chemistry conditions from these scenarios. Table 1 also provides an indication of how different types of surface waters within the region vary in terms of their relative sensitivity to Ni toxicity.

#### Effects assessment

An extensive effects database for chronic Ni toxicity to freshwater aquatic organisms was compiled in Europe under the existing substances regulations (EC 2008), and this was subsequently adopted for the derivation of the EQS for Ni in Europe (EU 2013). The database is primarily composed of data for temperate species, although some of the species represented within it are also relevant to tropical regions. A significant body of evidence was also available for assessing the bioavailability of Ni under different water chemistry conditions, which resulted in the development of a series of bioavailability models for Ni toxicity to algae, aquatic invertebrates, and fish. The bioavailability models were used to compile an approach for performing bioavailability normalizations of the entire SSD for chronic Ni toxicity in fresh waters (Nys et al. 2016).

A 2015 testing program resulted in chronic Ni toxicity data being collected for a number of tropical and temperate species in Australian natural waters (Peters et al. 2018), although the species tested were not necessarily relevant to the SEAM region.

In order to make a robust comparison of sensitivity between tropical and temperate organisms, a sufficient quantity of ecotoxicity data was needed. Conclusions from Gissi et al. (2016) and Binet et al. (2018) were that the currently available high‐quality chronic ecotoxicity data were insufficient to develop SSDs that were specific to tropical freshwater and marine ecosystems. To this end, laboratory toxicity testing was performed in a targeted fashion. An encompassing criterion for these data was that they needed to fulfill definitions of chronic endpoints because of their increasing use in global ERA frameworks, including Australian–New Zealand guidelines. Both aquatic and sediment compartments were addressed because of the relevance of both habitats to the exposure scenarios identified in the conceptual model. Likewise, both freshwater and marine organisms were tested.

#### Freshwater aquatic tropical ecotoxicity data

In terms of species selection for pelagic organisms, groups known to be sensitive from existing temperate databases were included to verify that their tropical counterparts within these groups were also sensitive. For example, the snail *Lymnaea stagnalis* and the crustacean *Ceriodaphnia dubia* are among the most sensitive species in the chronic freshwater Ni ecotoxicity database (Nys et al. 2016). To this end, tropical snails and crustaceans were identified as relevant test organisms. The minimum data requirements (MDRs) of global water quality guidelines for chemicals were also considered. Many MDRs specifically mention the need to include data for fish, and because no high‐quality chronic data were available for fish, the Asian tropical fish *Oryzias melastigma* was chosen for testing.

A comparative review of the sensitivity of tropical and temperate freshwater species to chronic Ni toxicity (Peters et al. 2019) considered differences in sensitivity at the ecosystem and individual taxa levels. The study by Peters et al. included that any differences in sensitivity between tropical and temperate ecosystems which were apparent from comparisons of SSDs were likely to be due to differences in the availability of test data between different climatic regions, as opposed to being related to differences in intrinsic sensitivity. Comparisons between the sensitivity of closely related taxa from different climatic regions did not reveal any differences that were greater than the level of variability observed between similarly related taxa from the same climatic region. The Peters et al. study, therefore, concluded that there was insufficient evidence to require separate SSDs for different climatic regions and that whole ecosystem protection would most likely be ensured by using the largest and most taxonomically diverse data set available, which would be achieved by combining all of the available reliable and relevant data for both tropical and temperate species into a single inclusive data set. For more information on the sensitivity of freshwater tropical and temperate species to chronic Ni toxicity, see Peters et al. (2019).

#### Marine aquatic tropical ecotoxicity data

Toxicity testing with pelagic marine organisms resulted in a substantial increase in the number of species in the tropical marine ecotoxicity databases. Wang et al. (2020) provided data for a tropical marine copepod, snail, and fish. Of these, the copepod *Tigriopus japonicus* was the most sensitive (EC10, 29 µg Ni/L), followed by the snail *Monodonta labio* (EC10, 34 µg Ni/L), and fish *Oryzias melastigma* (LC10, 1660 µg Ni/L) (Wang et al. 2020). Gissi et al. (2018) added data for 3 tropical marine invertebrates, the snail *Nassarius dorsatus*, the barnacle *Amphibalanus amphitrite*, and the copepod *Acartia sinjiensis*, with *Acartia* *sinjiensis* being the most sensitive tropical species tested to date, with an EC10 of 5.5 µg Ni/L. For comparison, EC10 and no observed effects concentration (NOEC) data for other tropical species in this data set (including those from Wang et al. 2020) ranged from 23 µg Ni/L (NOEC, sea urchin *Diadema savignyi*) to 3700 µg Ni/L (EC10, cyanobacteria) (Gissi et al. 2020). Data for tropical unicellular algae were generated, including *Tisochrysis lutea* (formerly *Isochrysis* sp.), *Ceratoneis closterium* (formerly *Nitzschia closterium*), and the free‐living stage of the coral zooxanthellae *Symbiodinium* sp. (Stauber et al. 2019). The conceptual model also identified coral reefs as critical structural components of tropical marine habitats that are likely to be exposed to Ni. No high‐quality chronic ecotoxicity data for Ni were identified for corals or their symbionts; this absence of data prompted a series of studies on different life stages of corals and on their microbiome. In a series of studies with multiple species of geographically relevant coral, data for coral fertilization (Gissi et al. 2017), effects of exposure to dissolved and particulate Ni on adult coral (Gissi et al. 2018; Gillmore et al. 2020), and effects to the coral microbiome (Gissi et al. 2019) were also added. Critically, these results showed that corals were not particularly sensitive to exposure to dissolved or particulate Ni. The end result of this testing yielded chronic data with sufficient numbers of species and a sufficiently diverse taxonomic coverage to assemble a stand‐alone tropical marine SSD.

Full results of aquatic toxicity tests for marine organisms exposed to Ni are reported in Gissi et al. (2017, 2018, 2019), Gillmore et al. (2020), and Wang et al. (2020).

Gissi et al. (2020) also showed that there were no significant differences between the sensitivity of marine tropical and temperate species to Ni, suggesting that tropical and temperate species could be combined in SSDs to maximize the taxonomic coverage for the derivation of marine Ni guideline values.

#### Benthic tropical ecotoxicity data

For marine benthic habitats, a multiple lines of evidence approach was adopted due to the absence of whole‐sediment bioassay protocols and tropical test species for generating ecotoxicological data relevant to the SEAM region (Gissi et al. 2016). The first line of evidence focused on the development of a sublethal whole‐sediment toxicity test for a tropical benthic marine organism endemic to the SEAM region and on generating effects data for sediments with different physicochemical properties (Stauber et al. 2019). Sediment data collected for the SEAM region can be found in Supporting Information SI4. For the present study, the juvenile life‐stage of the burrowing mud snail *Nassarius dorsatus* and the benthic diatom *C. closterium* were selected to provide representation of ecologically relevant taxa across different exposure scenarios. Neither of these species were found to respond to Ni at environmentally relevant concentrations and highlighted the difficulties with identifying relevant test species and developing standardized test procedures for use in risk assessment.

The second line of evidence used a standard temperate test species and protocol to investigate the relevance of chemical methods that can measure the bioavailable fraction of Ni to predict toxicity of Ni in sediments collected adjacent to Ni laterite mining operations in New Caledonia (Stauber et al. 2019). The species selected was the temperate epibenthic amphipod *Melita plumulosa* because it is a robust Australasian test species that is tolerant to a range of sediment types and sensitive to metal contaminants. The results of the Stauber et al. study found that the dilute‐acid extractable Ni concentration provided a reasonable estimate of toxicity in the field‐contaminated sediments and supported its use as a screening tool in sediment quality assessments rather than just the total recoverable Ni concentration, which was found to overestimate Ni toxicity of the field‐contaminated sediments. Both traditional chemical measurements suggested that differences in toxicity were predicted based on sediment type, which is consistent with conclusions from research on freshwater Ni sediment risk assessment (Besser et al. 2013; Schlekat et al. 2016). The Stauber et al. study also evaluated the diffusive gradients in thin‐films (DGT) technique and found it provided the best prediction of toxicity in the field‐contaminated sediments because it was able to integrate measurements of labile forms of Ni from the pore water, overlying water, and ingested sediment exposure pathways (Stauber et al. 2019). Consequently, the use of DGT allows prediction of toxicity that is independent of sediment type, which is in contrast to the traditional chemical measurements of potential Ni bioavailability. No differences in toxicity were predicted based on sediment type. Given the lack of standardized toxicity test protocols with ecologically relevant tropical species and limited sediment toxicity data available with which to derive sediment quality guideline values for the SEAM region, the derivation of a DGT‐based labile‐metal flux threshold could provide a cheaper and faster surrogate to ecotoxicological testing for the prediction of effects from sediment Ni exposure to benthic organisms.

The third line of evidence used a genomic approach called “metabarcoding” that utilizes environmental DNA (eDNA) to investigate changes in benthic community composition along a sediment Ni concentration gradient downstream of a large lateritic Ni deposit in New Caledonia (Stauber et al. 2019). Compositions of eukaryote, prokaryote, and diatom communities were determined at 10 sites along a gradient of sediment Ni concentrations ranging from 24 to 120 mg/kg dilute‐acid extractable Ni and 250 to 1500 mg/kg total‐recoverable Ni. The results of the Stauber et al. study revealed that the benthic eukaryote, diatom, and prokaryote community compositions changed significantly along the sediment Ni concentration gradient. Distance‐based linear models showed that, of the environmental predictor variables collected, the observed changes in community compositions correlated most strongly with the sediment Ni concentration gradient. Although community composition changes were found along the Ni contamination gradient, there was no observed decreasing gradient in biodiversity, evenness, or species richness of prokaryotes and eukaryotes. In other words, Ni contamination of sediments did not cause a significant decrease in the biodiversity as reflected by eDNA, even at sites closest to the mine discharges into the bay. However, diatom species richness and diversity did decrease as sediment Ni concentrations increased. This result suggests that species replacement may be occurring along the Ni concentration gradient. Threshold Indicator Taxa Analysis (TITAN) of the eukaryote data set revealed a shift from macrofaunal invertebrates at low Ni concentrations to microbial metazoans at higher Ni concentrations, whereas the prokaryote data set revealed a decrease in taxa from Actinobacteria and an increase in taxa from Chloroflexi and Firmicutes. Similar metal sensitivity and metal tolerance has been described for these phyla in other studies (Yin et al. 2015). Changes in community compositions alone, however, do not necessarily imply adverse ecological impacts, and further investigations into how these changes affect ecosystem health and function are required. Based on the response of taxa along the Ni concentration gradient, a threshold value of 46 mg/kg dilute‐acid extractable Ni was proposed to be protective for sensitive taxa (Stauber et al. 2019).

### Phase 3: Risk characterization and implementation of bioavailability

The effects assessments reached a conclusion that distributions of sensitivity to Ni were similar between tropical and temperate organisms for both marine (Gissi et al. 2020) and freshwater (Peters et al. 2019) systems. This conclusion supports an inclusive approach toward SSD compilation by combining all of the available reliable and relevant toxicity data regardless of climatic region, at least for chronic Ni toxicity.

An additional consideration that is relevant to Ni and other metals in fresh waters is that the data set should ideally enable bioavailability normalization by including only studies for which there is sufficient information about the water chemistry conditions in the tests to enable bioavailability to be accounted for. Ideally, the toxicity of Ni is determined under a range of water chemistry conditions in order to demonstrate the applicability of the bioavailability models (e.g., Schlekat et al. 2010; Peters et al. 2018). A study by Peters et al. (2019) found that data sets which enabled bioavailability normalization ensured a higher level of protection of the most sensitive waters because they allowed the results of tests which were conducted under less sensitive conditions to be corrected for higher sensitivity water chemistry conditions.

Limited data exist for the key water chemistry parameters that are required for performing bioavailability normalization. This means that there is also a very limited understanding of sensitivity of the local freshwater species within the SEAM region to Ni.

Different water chemistry scenarios (see Table 1) were used to derive site‐specific SSDs for a range of different water chemistries that are broadly representative of the diversity of fresh waters in the region, although with a tendency toward more sensitive conditions to minimize the potential for underprotection of local ecosystems. The SSDs included 36 species from both tropical and temperate conditions and represented the largest and most diverse Ni toxicity data set that could be normalized for local water chemistry conditions (Peters et al. 2019). A number of different thresholds were calculated, reflecting a range of potential levels of ecosystem protection (Table 1). The thresholds were derived to be protective of at least 95%, 90%, or 75% of species (i.e., HC5, HC10, and HC25) within the local ecosystems that the water chemistry scenarios represented.

Waters that are typified by high pH and low DOC concentrations exhibit the most sensitive conditions for Ni toxicity, that is, having the lowest HC5 values (Peters et al. 2018), and it is the lateritic geologies that tend to exhibit these water chemistry characteristics (Supporting Information SI3). The areas that are typified by limestone or volcanic geologies with circumneutral pH conditions exhibit a lower sensitivity to Ni toxicity due to a combination of having lower pH, higher DOC concentrations, and/or a higher hardness. The most sensitive lateritic waters are approximately 7 times more sensitive to Ni toxicity than the relatively insensitive neutral pH volcanic waters that are typified by much higher DOC concentrations. The slope of the SSDs is also relevant because the insensitive species are less affected by differences in bioavailability, and at the lower protection level of the HC25 (75% of species protected) the difference between the most and least sensitive water chemistry scenarios is reduced from a factor of 7 to a factor of 5.

The approach proposed for assessing the potential risks due to Ni in the SEAM region relies on existing information from a wide variety of different sources. It is inevitable that in some cases there will be differences in the findings and recommendations of the various information sources for a given aspect of the ERA. A multiple lines of evidence approach must be employed, which considers all of the available information in order to support the decisions made about issues such as the composition of ecotoxicity databases, the assessment and interpretation of exposure information, and the conclusions drawn.

The conclusions drawn from the ERA process should ideally be assessed against supporting information about local ecosystems, their uncertainties, and the extent to which they are affected by ambient Ni exposures. Existing Ni toxicity data (Peters et al. 2014; Hommen et al. 2015; Pereira et al. 2018; Nys et al. 2019) show that apical endpoints overestimate effects at the population and community levels, and that the HC5 values generated from apical endpoints are therefore conservative. This was also shown for the tropical ERA, where concentrations known to cause significant effects in laboratory tests showed no impact in terms of the biodiversity as revealed by eDNA within exposed ecosystems (Stauber et al. 2019). In areas like New Caledonia, where background levels of Ni are naturally elevated, organisms may have acclimated to these concentrations and developed tolerance, whereas laboratory‐cultured organisms with no previous exposure may not be representative in toxicity tests, or particularly sensitive species may not be present in local ecosystems due to potential Ni toxicity. Supporting information can come from a variety of different sources such as ecological assessments or field studies and may be especially helpful where several parallel assessments are performed along an exposure gradient (e.g., Stauber et al. 2019). Although these types of field‐based approaches are invariably more uncertain than laboratory‐based testing, they provide valuable insights into real ecosystems and how they can respond to exposures. This makes them a very valuable reference against which the conclusions of more abstract ERAs can be compared and validated.

#### Site‐specific EQS setting

Setting a relevant EQS requires an SSD of regionally relevant species with sufficient diversity to ensure that sensitive species are adequately protected, and an approach applied for correcting the ecosystem sensitivity to account for local differences in sensitivity due to differences in water chemistry. The 2 aims of regional relevance and sufficient diversity could be irreconcilable, especially if toxicity data are not available for a wide range of regionally relevant species. Maximizing the taxonomic diversity represented within the data set could potentially mean including some species that are not directly relevant to the region in question but could be considered as surrogates for relevant, but untested, species. The approach recommended by Peters et al. (2019) and Gissi et al. (2020) is to include all reliable and relevant studies, regardless of whether or not species are regionally relevant, in order to maximize the diversity of the SSD and to ensure adequate protection of potentially sensitive local species.

A study on Australian test species in field‐collected natural waters from Australia (Peters et al. 2018) concluded that the existing approaches for normalization of ecotoxicity data to local water chemistry conditions (e.g., Nys et al. 2016) were also appropriate for tropical species. Additionally, ongoing work in New Caledonia focuses on the development of an EQS in a region defined by high background Ni concentrations in fresh waters (e.g., tap water concentrations in the order of 10 µg/L; St‐Jean et al. 2018). The New Caledonia EQS project consists of a multistep approach and includes not only quantifying the background Ni concentrations but also expanding to selecting tests for waters with a wide range of bioavailability potentials of Ni, identifying and isolating native species for testing, and ultimately performing toxicity tests to generate reliable toxicity threshold values applicable to the area. Approaches and principles developed in the Nickel Environmental Risk Assessment program will be applied to the development of the EQS in New Caledonia.

## CONCLUSIONS

The present multiyear, multi‐institution research program was undertaken to migrate refined risk assessment approaches developed in North America, Europe, and Australia–New Zealand to the SEAM region. We developed an approach to determine if the individual components of classical ERA, including effects assessments, exposure assessments, and risk characterization methodologies (which includes bioavailability normalization), are applicable in this region.

A main finding of the present research program was that although ecosystems and exposures may be different in tropical systems, ERA paradigms are constant. A large chronic ecotoxicity data set for Ni is now available for tropical species, and we found that tropical ecosystems are not uniquely sensitive to Ni exposure; hence, scientific support exists for combining tropical and temperate data sets to develop tropical EQSs.

Exposure databases have been developed but remain incomplete in most areas. However, the compiled database was sufficient to allow for selection of “typical waters” for fresh waters in the SEAM region (based on catchment geology). The approach is conservative and may lead to the conclusion that additional site‐specific data are required in order to confidently determine or rule out conclusions of risk. Additional water chemistry data will be required from the sites to more accurately define Ni exposures and assess Ni bioavailability. The bioavailability relationships observed in temperate systems appear to be consistent for tropical habitats, and tiered risk refinement options are now available, such as site‐specific bioavailability assessment, localized or site‐specific SSD, or direct assessment of ecological effects.

In developing EQS values for the SEAM region, high background Ni concentrations must be considered. A one‐size‐fits‐all approach will not work in unique areas like New Caledonia and other Ni laterite districts that have high natural background concentrations of Ni. Temperate and tropical threshold values cannot be directly applied, and additional investigation of the habitats and species therein is warranted. The generic tropical database can be used as a starting point to examine the unique biotic and abiotic characteristics of a specific ecosystem, for example, New Caledonia. By demonstrating that the constructs of traditional ERA can be applicable in other ecosystems, we have developed the building blocks of a tiered approach that can be used for performing ERA in any region.

## DISCLAIMER

The authors declare no conflicts of interest.

## SUPPORTING INFORMATION

**SI1.** Guideline values summary for the SEAM region.

**SI2.** Example of a Leopold matrix for decision making for research in the SEAM region.

**SI3.** SEAM water chemistry and sediment data set.

## Supporting information

This article contains online‐only Supporting Information.

Supporting information.

Supporting information.

Supporting information.

## Data Availability

Data associated with this manuscript are available in the Supporting Information and/or are available from the authors of the paper. Requests for data should be directed to the corresponding author, Emily Garman (egarman@nipera.org).
