# Supplementary material for: Development of a bioavailability‐based risk assessment framework for nickel in Southeast Asia and Melanesia
Source: Integr Environ Assess Manag. 2021 Feb 8;17(4):802–13. doi: 10.1002/ieam.4384 (PMC8359217; doi:10.1002/ieam.4384)
Supplement: Supplementary file 2 — Supporting information. [file IEAM-17-802-s003.pdf]

Supplemental Information File 2. Example of a Leopold Matrix for future tropical ERA research prioritization

A modified Leopold Matrix based on uncertainty, importance, and practicality can be used to prioritize research that is both impactful and achievable. The approach relies on expert opinion to identify environmental compartments with gaps and to rank them accordingly on a scale of 1 to 5 for: Amount of certainty/data availability (1=lowest and 5=highest); Relative importance/impact (1=lowest, 5=highest), and Practicality (1=hardest and 5=easiest). The sum of the three assessment scores can be used to rank priorities for research needs.

Compartment		Uncertainty<br>1=low, 5=high	Tropical importance<br>1=low, 5=high	Practicality (within 5 yrs)<br>1=hard, 5=easy	Total<br>score
Marine	Seagrass	4	4.5	2.5	11
	Mangroves	4	4.5	2.5	11
	Deep sea	5	4	1	10
Estuarine	Pelagic	3.5	4	4	11.5
	Sediments	3.5	4	3	10.5
	Suspended sediments	3.5	4	3	10.5
Freshwater	Floodplain	4	4	1.5	9.5
	Sediments	3	3.5	4.5	11
	Pelagic	1.5	3.5	4.5	9.5
	Wetlands	4	4	2	10
Terrestrial	Soil organisms	4	4	5	13
	Soil processes	4	3.5	5	12.5
Groundwater	Stygofauna	5	2.5	1	8.5

Level of priority
High
Medium
Low
